# Pericardial Cyst: Never Too Late to Diagnose

**DOI:** 10.3390/jcm7110399

**Published:** 2018-10-30

**Authors:** Katrina Lennon Collins, Fady Zakharious, Amit K. J. Mandal, Constantinos G. Missouris

**Affiliations:** 1 Departments of Cardiology and Internal Medicine, Frimley Health National Health Service (NHS) Foundation Trust, Wexham Park Hospital, Slough, Berkshire SL2 4HL, UK; katrina.lennon@nhs.net (K.L.C.); f.zakharious@nhs.net (F.Z.); amit.mandal@nhs.net (A.K.J.M.); 2 Department of Cardiology, University of Cyprus Medical School, Nicosia 1678, Cyprus

**Keywords:** congenital heart disease, elderly, pericardial cyst, imaging, asymptomatic, conservative management

## Abstract

Pericardial cysts are uncommon benign lesions of the middle mediastinum, making up less than 6% of all mediastinal masses. They are often detected as incidental findings on chest imaging and some can resolve spontaneously. Rarely, however, they may cause symptoms of chest pain, right ventricular outflow obstruction, and persistent cough. Furthermore, they may affect cardiac tamponade after acute rupture or cyst haemorrhage resulting in sudden death. We report the case of a 102-year-old woman presenting with urosepsis, in whom routine chest radiography was initially suspicious of advanced bronchial carcinoma. Further imaging supported a diagnosis of one of the largest pericardial cysts described in the literature located in the right parahilar space. The patient was appropriately managed conservatively.

## 1. Introduction

Pericardial cysts are rare benign, congenital lesions found in the middle mediastinum, usually measuring less than 5 cm. In the majority of patients they remain clinically dormant and are detected in middle age as incidental findings on chest radiography. Rarely however, they may cause symptoms of chest pain and dyspnoea or lead to life threatening complications resulting from obstruction or compression of associated anatomical structures. We report the case of an asymptomatic 102-year-old lady who was found to have a one of the largest pericardial cysts described in the literature located in the right parahilar space.

## 2. Case History

A 102-year-old woman with no relevant past medical history presented to our service in a toxic state, with rigors and dysuria. Her temperature was 38.4 °C, the pulse was 117 beats per minute and the supine blood pressure 87/63 mmHg. Clinical examination was otherwise unremarkable. The resting 12 lead electrocardiogram revealed sinus tachycardia. Routine haematology and biochemistry revealed a haemoglobin of 13.7 g/dL, white blood cell count of 11.5 × 10^9^/L, C-reactive protein of 100 mg/dL and estimated glomerular filtration rate (eGFR) at 46 mL/min/1.73 m^2^. Urinalysis was strongly positive for infection with blood cultures subsequently growing *Escherichia coli* from one bottle.

**Figure 1:**
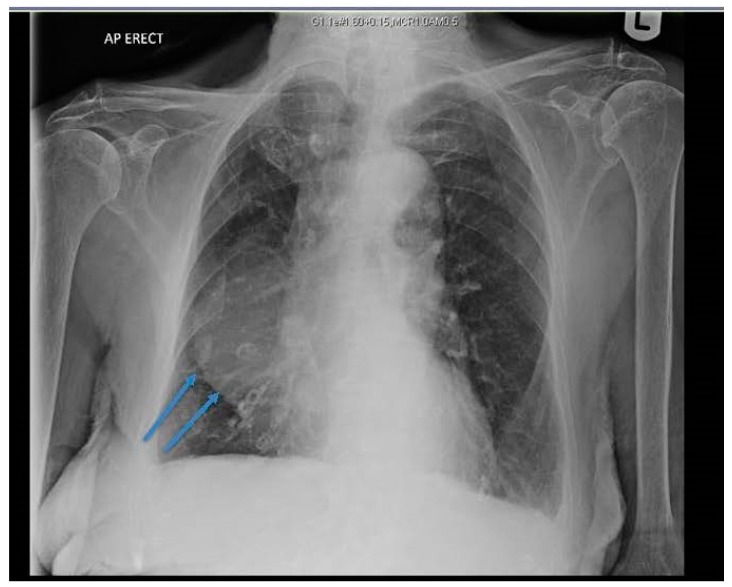
Chest X-ray (CXR) showing right sided soft tissue density, hemispherical in shape, projected over the right middle and lower zones (arrows).

**Figure 2:**
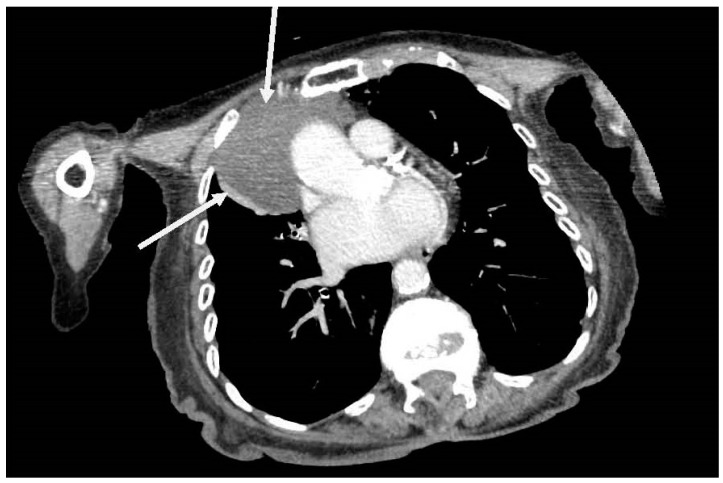
Axial computerised tomography (CT) showing an 8 cm × 5 cm low attenuation right sided soft tissue mass arising from pericardium and abutting the anterior mediastinum and anterior chest wall (arrows).

The chest X-ray (CXR) revealed a well-defined hemispherical soft tissue density, projected over the right parahilar region, as shown in Figure 1. No previous CXR was available for comparison. Computerised tomography (CT) of chest, abdomen, and pelvis with contrast demonstrated an 8 cm × 5 cm low attenuation lesion abutting the pericardium, the anterior mediastinum, and anterior chest wall, as shown in Figure 2. The findings were consistent with a large pericardial cyst and no bronchogenic malignancy was identified. Bronchiectatic changes in the middle lobe were consistent with longstanding compression. The patient was treated with intravenous co-amoxiclav and made an uneventful recovery.

In light of the absence of symptoms, current wellbeing, and extreme advanced age of the patient, it was agreed not to pursue any further intervention or complex imaging. She returned to the retirement home 5 days after admission to hospital, and on review in the outpatient clinic 2 months hence remains asymptomatic.

## 3. Discussion

Pericardial cysts are rare benign congenital lesions of the mediastinum that develop as a result of failure of fusion of one of the mesenchymal lacunae that form the pericardial sac. This leads to metamorphosis into a cyst wall composed of a thin layer of fibrous tissue lined with a single layer of mesothelial cells. The majority are found in the right anterior costophrenic angle (70%) [1,2], but can sit anywhere in the mediastinum adjacent to the heart. The reported incidence is 1:100,000 patients [1,2,3,4,5], although this is likely to be an underestimate as over 50% remain clinically silent [3] and are usually identified incidentally on imaging for an unrelated clinical condition. More than 60% of pericardial cysts are detected between the third and fifth decades of life. As far as we are aware, we record here the oldest patient found to have a pericardial cyst.

Pericardial cysts are typically less than 5 cm in diameter [1]. Larger cysts are associated with attributable symptoms, including chest pain, cough, dyspnoea, and dysphagia. Potentially fatal complications arise from cyst rupture/haemorrhage, erosion, and compression involving associated structures. These include cardiac tamponade, right ventricular outflow tract obstruction, superior vena cava syndrome, and obstruction of the right main stem bronchus [1,2,3,4,5]. Principle differentials alongside bronchogenic neoplasm are pericardial fat pad, thymoma, mediastinal teratoma, Morgagni hernia, and any simple cystic mediastinal masses.

CT with contrast and Cardiac Magnetic Resonance Imaging (CMR) are the imaging modalities of choice to delineate the aetiology and tissue characterization [4]. Transthoracic or transoesophageal echocardiography is widely used to assess the haemodynamic significance of the cyst with respect to right ventricular outflow obstruction or constrictive physiology to the right or left ventricles [4].

Management is dependent on the presence and or severity of symptoms. Pericardial cysts have been known to spontaneously resolve. The mechanism by which this occurs is postulated to be through cyst rupture [5]. In our patient, conservative management was justified because of the absence of symptoms and advanced age.

In symptomatic patients, surgical excision is the definitive treatment, either via thoracotomy, sternotomy, or video-assisted thoracic surgery (VATS) [2]. Cyst aspiration may provide a therapeutic bridge, but re-amassment of the lesion is seen in 30% of cases. Asymptomatic cysts should be monitored to assess for growth and structural change [1,3].

Our case report clearly highlights that pericardial cyst can present as an incidental finding in asymptomatic elderly patients which may lead to misdiagnosis of more sinister pulmonary pathology. In addition to plain chest radiography, CT or CMR are the diagnostic tools of choice to allow an appropriate management path thereafter. In younger patients, however, emphasis is laid on the importance of careful monitoring and expectant observation for subtle changes in physiology or haemodynamics with cardiac imaging modalities to assess for any enlargement that may compromise the heart or cause obstruction to nearby structures.

## 4. Key Points

- Pericardial cysts are uncommon benign lesions of the middle mediastinum and are often detected by chance.
- They may mimic more sinister mediastinal pathology.
- The clinical course is variable, from asymptomatic to sudden death from cardiac tamponade, and emphasis is laid on the importance of expectant surveillance.

## References

[1] Patel J., Park C., Michaels J., Rosen S., Kort S. (2004). Pericardial cyst: Case reports and a literature review. Echocardiography.

[2] Najib M.Q., Chaliki H.P., Raizada A., Ganji J.L., Panse P.M., Click R.L. (2011). Symptomatic pericardial cyst: A case series. Eur. J. Echocardiogr..

[3] Lau C.L., Davis R.D. (2004). Chapter 56: The Mediastinum. Sabiston Textbook of Surgery.

[4] Yared K., Baggish A., Picard M.H., Hoffmann U., Hung J. (2010). Multimodality imaging of pericardial diseases. JACC Cardiovasc. Imaging.

[5] Kruger S.R., Michaud J., Cannom D.S. (1985). Spontaneous resolution of a pericardial cyst. Am. Heart J..

